# Therapeutic Drug Monitoring for Biologic and Small-Molecule Therapies for Inflammatory Bowel Disease

**DOI:** 10.3390/medicina60020250

**Published:** 2024-01-31

**Authors:** Krishneel Dutt, Abhinav Vasudevan

**Affiliations:** 1 Eastern Health, 8 Arnold Street, Box Hill, VIC 3128, Australia; kris.dutt@monash.edu; 2 Eastern Health Clinical School, Monash University, 8 Arnold Street, Box Hill, VIC 3128, Australia

**Keywords:** inflammatory bowel disease, therapeutic drug monitoring (TDM), pharmacokinetics, ustekinumab, vedolizumab, risankizumab, JAK inhibitors, tofacitinib, upadacitinib, filgotinib, ozanimod

## Abstract

*Background:* Inflammatory bowel disease (IBD), encompassing ulcerative colitis and Crohn’s disease, necessitates long-term medical therapy to manage symptoms and prevent complications. Therapeutic drug monitoring (TDM) has emerged as a strategy to optimize treatment efficacy, particularly with anti-tumour necrosis factor (anti-TNF) alpha drugs. This review explores the role of TDM for non-anti-TNF advanced therapies in IBD, focusing on vedolizumab, ustekinumab, tofacitinib, upadacitinib, risankizumab and ozanimod. *Methods:* The literature search, conducted through OVID (Medline) and PubMed, delves into proactive versus reactive TDM, timing of monitoring and methods for measuring drug levels and anti-drug antibodies. *Results:* While ustekinumab and vedolizumab exhibit exposure–response relationships, consensus on target levels and the role of TDM adjustments remains elusive. Limited data on risankizumab suggest a dose-dependent response, while for small molecule therapies (janus kinase inhibitors and ozanimod), the absence of real-world data and commercially available TDM tools pose challenges. *Conclusion:* At present, with the available data, there is a limited role for TDM in non-anti-TNF biologic and small-molecule therapies. This review underscores the need for further research to delineate the utility of TDM in guiding treatment decisions for these agents.

## 1. Introduction

Inflammatory bowel disease (IBD), including ulcerative colitis and Crohn’s disease, is an immune-mediated disorder primarily involving the gastrointestinal tract and often requiring long-term medical therapy to prevent complications and reduce the risk of hospitalisation or surgery. Multiple therapeutic agents have been developed for treating IBD, and there is a growing armamentarium of advanced therapies targeting different aspects of the inflammatory cascade that have been shown to be safe and efficacious in treating both Crohn’s disease and ulcerative colitis. With the advent of more agents, there has also been a greater focus on achieving more stringent disease control, with a resulting need to optimise therapies being used to ensure that the maximal clinical benefits are achieved [1]. Therapeutic drug monitoring (TDM) is one such method for optimising therapies. The role of TDM in adjusting treatment with anti-tumour necrosis factor (anti-TNF) alpha drugs, such as infliximab and adalimumab, is well established and can lead to higher rates of clinical response [2]. Overall, therapeutic drug monitoring in certain populations can allow for lower rates of adverse events, disease progression, complications and a reduction in the need for surgery [3]. Utilising TDM to guide treatment decisions for patients who are not responding adequately to therapy has become an established part of treatment with anti-TNF therapy in inflammatory bowel disease [4].

The role of therapeutic drug monitoring with non-TNF advanced therapies in inflammatory bowel disease is less well established. Intuitively, monitoring drug levels with these therapies should provide an indication of therapeutic effect, but there is complexity associated with pharmacologic properties of biologic and small-molecule therapies so that broad generalisations on the role of therapeutic drug monitoring cannot be established and a specific approach is needed for each agent. This review aims to discuss the role of therapeutic drug monitoring for non-anti-TNF biologic and small-molecule therapies in the treatment of IBD. This review will focus on issues relating to the timing and measurement of drug levels, as well as drug-specific factors that may be important when considering the role of TDM. Advanced therapies that are currently available for the treatment of IBD will be covered, specifically, vedolizumab, ustekinumab, tofacitinib, upadacitinib, risankizumab and ozanimod. 

## 2. Methods

This narrative literature review was conducted by identifying the relevant literature through searches via OVID (Medline) and PudMed. It encapsulated the literature up until the 31 November 2023. Keywords used to identify studies included “therapeutic drug monitoring”, “inflammatory bowel disease” and individual treatments as per the aim of this review. The literature search was expanded to include pharmacokinetics, exposure–response, drug assay and ADA monitoring, and these terms were also sought in separate searches with each individual therapeutic agent. Studies were included based on the relevance to pharmacokinetics, pharmacodynamics and therapeutic drug monitoring of each biologic and small-molecule therapy reviewed. Relevant references from identified articles were also identified and used as a part of this narrative review.

## 3. Results

### 3.1. Factors to Consider with Therapeutic Drug Monitoring

#### 3.1.1. Proactive vs. Reactive Therapeutic Drug Monitoring

Therapeutic drug monitoring can take two forms: proactive, in which drug levels are measured at predetermined points in time, or reactive, where measurements are taken in response to active disease. The evidence for proactive TDM indicates that it may be of greater benefit than reactive TDM, specifically for anti-TNF therapy. In a simulated model of patients receiving infliximab monotherapy, proactive monitoring was shown to lead to higher drug retention, lower anti-drug antibodies and lower rates of flares [5]. A meta-analysis by Shah et al. suggests that proactive TDM may provide a cost-effective method to prevent treatment failure, specifically when combined with appropriate de-escalation [6]. Reactive monitoring, though, does lead to better outcomes compared to no TDM [7,8]. It appears to adhere to cost reduction compared to blind dose escalation in patients with presumed loss of response [9] and improved biochemical and endoscopic healing [10]. 

Overall, therapeutic drug monitoring appears to be cost-effective and may improve clinical and patient outcomes [11].

#### 3.1.2. Timing of Therapeutic Drug Monitoring

Available data suggest the serum trough levels and anti-drug antibodies (ADAs) correlate with clinical and endoscopic response to anti-TNF agents [12,13,14] ustekinumab [15] and vedolizumab [16,17]. Proactive therapeutic drug monitoring at the time of induction is likely to reduce the risk of primary non-response, development of immunogenicity and provide longer-term clinical benefits [18]. The role of proactive TDM has been demonstrated mainly with anti-TNF agents [7,19,20,21]; however, data also support proactive TDM during vedolizumab induction [22,23]. A large portion of studies have evaluated utilising TDM while on maintenance therapy. There have been studies suggesting there is no benefit from therapeutic drug monitoring when treating IBD, including the TAILORIX [24] trial. In this randomised controlled trial, individuals were randomised into three groups: group 1 had 2.5mg/kg dose increases up to two times based on CDAI or infliximab level, group 2 had 5 mg/kg dose increases up to one time based on the same criteria, and group 3 had 5 mg/kg dose increases based on CDAI alone. This study showed no significant difference between escalation using trough levels compared to symptoms alone. This study had noteworthy limitations, including escalating doses in patients without objective evidence of disease activity and dose escalation prioritised by CDAI scores. More recent trials demonstrated positive benefits of therapeutic drug monitoring, including the PAILOT, a paediatric study demonstrating a proactive escalation of adalimumab led to higher rates of steroid remission compared to a reactive model of escalating at the time of loss of response, and PRECISION, a randomised controlled trial which supports dose escalation of infliximab based on trough levels to lead to lower rates of loss of response compared to no-dose escalation [25,26]. Other biologic therapies not targeting anti-TNF have demonstrated an exposure response which supports the idea that higher drug concentrations lead to increased clinical response [15,22,27]; however, the role of proactive TDM and dose adjustment of therapies with these agents are not well established.

#### 3.1.3. Methods of Measuring Drug Levels and Anti-Drug Antibodies (ADAs)

There are multiple methods for therapeutic drug monitoring, including enzyme-linked immunoassays (ELISAs), liquid chromatography and radioimmunoassay, with ELISA being the most common in commercial use [28,29,30,31,32]. The key components of these assays are summarised in Table 1. Overall, variability between assays has been compared and appears to be negligible, though it is advised to use the same drug level assay for TDM, allowing for greater consistency and reduction in variability in real-world practice [33]. 

ADA testing is commonly achieved via quantitative sandwich ELISA, using a monoclonal ADA as the calibrator. This method, however, is sensitive to the serum drug concentrations as these can interfere with the measurement of anti-drug antibodies by the assay, so low serum levels of the drug are required to obtain an accurate assessment of anti-drug antibody levels [34]. Radioimmunoassay and mobility shift assays have lower rates of cross-reactivity with serum drug concentrations and can provide more accurate assessments of the levels of anti-drug antibodies even when serum drug levels are higher, although these assays can have higher financial and technical costs [35,36].

## 4. Advanced Therapies Using Therapeutic Drug Monitoring

### 4.1. Monoclonal Antibodies

#### 4.1.1. Ustekinumab

Ustekinumab, an interleukin (IL) 12/23p40 antagonist, can be used in the treatment of both Crohn’s disease and ulcerative colitis [37,38]. It exerts its effect by neutralising IL-12 and IL-23, thereby downregulating several pro-inflammatory mediators including interleukin 8, TNF-α and monocyte chemoattractant protein-1 (MCP-1) [39]. It is usually given as an initial intravenous dose of treatment followed by maintenance subcutaneous treatment every 8 or 12 weeks. 

##### Pharmacokinetics and Pharmacodynamics

In a two-compartment model, peak serum concentration of ustekinumab occurs one hour after IV administration [40]. Steady state concentrations are reached 16 weeks post induction, at the time of the second maintenance dose [40]. 

Subcutaneous bioavailability of the drug is within the range of 78–88% [27,40]. Similar to other biologic therapies discussed later, clearance of ustekinumab increases with lower albumin levels and higher C-reactive protein (CRP) levels [27].

Exposure–response modelling suggests that patients with a higher baseline CRP are likely to have a better response to ustekinumab during induction [27,41]. Intravenous re-induction with ustekinumab seems to cause similar drug exposure to the initial induction, with steady state achieved 16 weeks after re-induction [27].

Immunogenicity was noted to be low in the pivotal phase 3 trials for ustekinumab in both UC and CD [40]. A systematic review estimated the rate of ADA development on maintenance therapy to be between 4.2% and 5.6% [42]. It has been suggested that immunogenicity is transient with ustekinumab [40]; however, a recent study has shown that it may correlate to loss of response [43]. The use of immunomodulators, such as thiopurines or methotrexate, is not thought to have significant impacts on serum drug concentrations or ADAs [27,40].

##### Therapeutic Drug Monitoring

Available data on the correlation between trough serum ustekinumab drug concentrations and clinical outcomes have been mixed, making the role of therapeutic drug monitoring less clear. Multiple studies, though, have shown an association between higher drug levels and better clinical and biochemical response to ustekinumab [15,44,45,46,47]. The majority of these studies evaluated serum trough levels via ELISA at the time of ustekinumab induction or mobility shift assay and at follow-up periods up to week 26. The identified target trough levels required on maintenance therapy to achieve a clinical response have varied substantially [42], with levels as low as 1.3 µg/mL [40] and up to levels of 4.5 µg/mL [15] being observed as targets to establish clinical response. The timing of drug level measurement is also likely to have an impact on these reported drug levels and may explain some of the variation in identified cut-off values [46]. Drug levels should ideally be measured prior to the second maintenance dose as ustekinumab reaches steady state concentrations [40,46]. The substantial variability in levels observed may partly relate to differences in the methods of reporting trough concentrations between studies, as well as the assays used to measure drug levels. Nonetheless, this variability reflects the imprecise nature of using a dedicated cut-off value when using ustekinumab therapy to guide treatment doses [48,49].

Not all studies have shown an association between clinical response and ustekinumab levels. Painchart et al. prospectively evaluated drug levels and associations with clinical response in Crohn’s disease [50]. They observed a cohort of patients on maintenance ustekinumab and an induction cohort who were just commencing ustekinumab treatment. Ustekinumab trough levels using an ELISA were collected after each dose of therapy. In their prospective study, no significant difference in median ustekinumab trough levels were noted between responders and non-responders post induction and at 6 months post commencement. In the maintenance cohort, similarly, no difference was noted between clinical response and median ustekinumab trough levels [50]. 

A novel study looking at tissue ustekinumab and IL-23 similarly found no significant correlation between levels and clinical response in Crohn’s disease [51]. Serum concentrations of ustekinumab correlated with tissue concentrations in this study. However, the ratio of IL-23-to-ustekinumab levels correlated with histological inflammation, suggesting that the ability of ustekinumab to suppress IL-23 levels may have a role in reducing the inflammatory burden.

Given the variability in reported cut-off levels for ustekinumab, despite the apparent association between higher levels and better outcomes, it seems that a more nuanced approach to utilising levels to guide clinical practice is needed, where the trough values are taken in the clinical context rather than as an absolute cut-off. For example, monitoring for an increase in trough ustekinumab levels following dose escalation of ustekinumab compared to levels taken prior to escalation of therapy can be useful in determining the likelihood of response to therapy, with higher ustekinumab levels achieved in patients who achieve both clinical and endoscopic remission compared to those who do not achieve these outcomes [52].

#### 4.1.2. Vedolizumab

Developed primarily for the treatment of IBD, vedolizumab is an integrin inhibitor against the α4β7 integrin involved in leukocyte trafficking in the gastric luminal tract. This occurs via interference in α4β7 integrins interaction with the mucosal vascular addressin cell adhesion molecule 1 (MAdCAM-1) [53]. The anti-inflammatory effect of vedolizumab appears to extend beyond this to include changes in the innate immune system, including changes in macrophage populations in the luminal tract and alteration in chemokine and cytokine responses [54]. 

##### Pharmacokinetics and Pharmacodynamics

Vedolizumab is available for use in intravenous and subcutaneous formulations. The intravenous regime requires two induction doses of 300 mg at week 0 and 2, followed by maintenance dosing at 300 mg eight times weekly. The subcutaneous formulation also requires the same induction regime, followed by 108 mg of subcutaneous drug administered every 2 weeks.

Vedolizumab, like other monoclonal antibodies, has a complex clearance mechanism. The molecule is metabolised into peptides and amino acids, which are excreted in urine [55]. Predictors of accelerated clearance include low albumin and an elevated body weight [56]. Data suggest that clearance may also be influenced by inflammatory burden in ulcerative colitis [57].

In vitro studies have suggested that there is complete saturation of α4β7 integrin receptor after a single or multiple doses of vedolizumab when the drug was detectable in serum [56]. Immunogenicity occurs with vedolizumab, although the reported rates are lower than with anti-TNF therapy (estimated to be between 1 and 4% of patients during induction) [57]. The initial GEMINI trials noted nine patients had anti-drug antibodies during the maintenance phase, with none of this subgroup achieving clinical response [58]. Immunomodulators may have a role in reducing the immunogenicity of vedolizumab [57,58,59]. Immunogenicity may also be a transient phenomenon, with observed reductions in autoantibodies during the maintenance phase [60]. The clinical relevance of anti-drug antibodies to vedolizumab has been debated, with some studies suggesting that co-immunomodulator therapy does not improve clinical outcomes [61,62], but it has been suggested that the development of anti-drug antibodies to vedolizumab can lead to primary non-response [58,59], so the use of concomitant immunomodulator therapy varies between clinicians.

Subcutaneous formulations have a similar pharmacokinetic profile to intravenous preparations [63]. Notably, subcutaneous formulations achieve higher trough levels compared to vedolizumab, due to a shorter dosing interval. Similar rates of immunogenicity are observed in patients using subcutaneous formulations to intravenous therapy [63].

##### Therapeutic Drug Monitoring

Several studies have assessed the exposure–response relationship of intravenous vedolizumab to attempt to determine a therapeutic drug target during induction and maintenance therapy. Absolute cut-off trough levels to achieve clinical response has varied widely across studies, ranging from 14 µg/mL to 28 µg/mL during the induction phase [17,57,64]. Singh et al. performed a systematic review of vedolizumab and found trough levels >18–20 µg/mL during induction (at week 6) and >10–12 µg/mL during maintenance therapy were associated with higher rates of clinical response [16]. A recent large prospective study showed marked interindividual variability in trough levels, with a large proportion of patients (39%) being below the expected trough level of 14 µg/mL at week 6, despite showing clinical response [65]. There was no significant difference across the measured quartiles, grouped as those with trough levels <10 µg/mL, 10.1–16.6 µg/mL, 16.7–23.3 µg/mL and >23.4 µg/mL [65]. Though it should be noted the lower quartile (<10 µg/mL) had a lower clinical response rate compared to higher quartile drug trough levels, although this did not reach statistical significance. The wide range of these levels makes it difficult to discern what specific trough level to target, but generally levels below 10 µg/mL during maintenance therapy would be considered subtherapeutic. Studies assessing optimising therapy in these patients and correlating this with clinical outcomes are needed.

Multiple studies have identified an exposure–response relationship to vedolizumab during induction and in the early post-induction phase [22,66,67]. Though this relationship exists, a definitive absolute value to target at each phase of treatment is difficult to define given the variability amongst defined values in studies. An estimation, based on the given data, would suggest a target level for vedolizumab would likely lie between 20 and 30 µg/mL in the induction period and between 12 and 18 µg/mL during maintenance.

Overall, there appears to be an exposure–response relationship to vedolizumab in the treatment of IBD. Whether higher levels reflect patients who are more likely to respond or whether adjusting the dose of therapy will improve outcomes requires further investigation. The heterogeneity in the study populations and the variability in reporting of therapeutic targets between quartiles and absolute values further add to the difficulty in translating these data to clinical practice.

#### 4.1.3. Risankizumab

IL-23 is a known mediator of the pro-inflammatory response in inflammatory bowel disease. Risankizumab is an IL-23 antagonist, with proven efficacy as an induction and maintenance treatment in Crohn’s disease and ulcerative colitis [68,69].

##### Pharmacokinetics and Pharmacodynamics

Risankizumab requires three intravenous induction doses (600 mg at weeks 0, 4 and 8), followed by eight weekly subcutaneous injections. Maintenance dosing varies, with both 180 mg and 360 mg eight times weekly approved for use in both Crohn’s disease and ulcerative colitis. Risankizumab has linear pharmacokinetics with dose-proportionate increases in levels observed with repeated administration. Steady state concentrations are achieved 16 weeks after commencement of standard induction [70]. Factors increasing drug clearance in patients with Crohn’s disease include low albumin, high faecal calprotectin levels and corticosteroid use at the time of induction [71].

The impact of immunogenicity on plasma concentrations appears negligible with risankizumab. In patients with psoriasis, the incidence of anti-drug and neutralising antibodies was estimated to be between 1 and 3% [70,72]. In these studies, the presence of anti-drug antibodies was associated with increased drug clearance and lower detectable serum levels. A similar proportion of ADAs were detected in Crohn’s disease patients but had no apparent effect on drug clearance [71]. This may be explained by higher doses of risankizumab being administered and due to concomitant immunomodulation therapy use in the Crohn’s cohort. 

Dose-response data suggest that higher doses achieve greater clinical efficacy [69,71], hence the 360 mg suggested dose for Crohn’s. There are no data at present on the effect-response at higher or more frequent dosing.

##### Therapeutic Drug Monitoring

Exposure–response analysis from the pivotal studies of risankizumab suggest greater clinical and endoscopic remission rates with higher doses of treatment [68,69,71]. This dose-dependent response plateaus for induction doses greater than 600 mg at week 12 and maintenance doses over 360 mg at week 52. It is expected that with higher doses average serum concentrations would rise, though the impact on clinical and endoscopic outcomes would be marginal based on this modelling.

There are limited data on therapeutic drug monitoring with risankizumab. Currently, prospective trials in both Crohn’s disease and psoriasis are assessing the association between drug monitoring and clinical response [73,74], though no data were available at the time of this review.

### 4.2. Small-Molecule Therapy

#### 4.2.1. Janus Kinase (JAK) Inhibitors

Three JAK inhibitors are currently approved for use in IBD: the pan-JAK inhibitor tofacitinib and JAK-1 selective inhibitors upadacitinib and filgotinib. Upadacitinib is efficacious in both Crohn’s disease and ulcerative colitis [75,76], while tofacitinib and filgotinib are only efficacious in ulcerative colitis [77,78].

##### Pharmacokinetics and Pharmacodynamics

The immune effects of the JAK pathway involve the transcription of multiple cytokines, interferons and growth factors. JAKs comprise four distinct entities: JAK1, JAK2, JAK 3 and tyrosine kinase 2 [79]. They are located within the cytoplasmic domain of multiple cytokine receptors such as interferon-γ, interleukin-2, interleukin 6 and interleukin 12/23. Upon activation, phosphorylation of the JAKs molecules and the subsequent interaction with the signal transducer and activators of transcription (STAT) initiate the response. The phosphorylated STAT molecules translocate to the cell nucleus, binding to promoter regions [80]. This results in transcription of proinflammatory mediators, which leads to lymphocyte proliferation, humoral immune responses and T-cell differentiation [81]. 

Tofacitinib is a rapidly absorbed agent with peak concentration reached within 1 h of administration [82]. Its half-life is approximately 3 h. CYP3A4 and CYP2C19 are implicated in the metabolism of tofacitinib [82]. Bioavailability is estimated to be around 75% after oral administration [83].

Upadacitinib is metabolised by the same hepatic cytochrome P450 pathway. It reaches peak plasma concentrations within 1-2 h and has a half-life of approximately 4 h [84].

Filgotinib has similar pharmacokinetic properties, though its half-life is slightly longer, approximately 5 h, and peak plasma concentration is reached slightly later, between 1–5 h post administration [83].

##### Therapeutic Drug Monitoring

Population pharmacokinetic monitoring has suggested greater clinical efficacy for tofacitinib with higher doses [85]. Higher doses are also noted to produce a greater clinical response in patients with prior anti-TNF exposure [86,87].

For upadacitinib, it appears that induction dosing at 45 mg daily and maintenance dosing at 30 mg daily achieves maximal clinical and endoscopic healing [88,89]. The exposure-response appears to plateau above 45 mg daily during induction and over 30 mg daily during maintenance therapy. There is some suggestion that extending the induction phase to 16 weeks instead of 12 may result in clinical response being achieved in some people who otherwise failed to reach an adequate response after 8 weeks of induction therapy [90].

Exposure–response data for filgotinib show a similar pattern to other JAK inhibitors. Meng et al. demonstrated a plateau in the phosphorylation of JAK-related interleukin-6-induced STAT-1 at advised maintenance doses (200 mg) of filgotinib. At these doses, based on the phase 2b/3 trials, there was a 75–80% clinical response rate at this exposure rate on modelling [91].

Overall, in clinical practice, the role of TDM for JAK inhibitors remains unclear. Currently, there are no available drug assays to allow for reliable testing. Dose escalation overall will likely only lead to marginal gains in clinical and biochemical response. Adverse events increase as the dose of therapy increases, so the role of therapeutic drug monitoring in guiding dose escalation of therapy requires further research [92].

#### 4.2.2. Ozanimod

Ozanimod, a sphigosine-1-phosphate (S1P) receptor modulator, has been shown to be efficacious in the treatment of ulcerative colitis [93]. The S1P receptor exhibits a proinflammatory effect through its involvement in lymphocyte migration in the gastrointestinal tract [94].

##### Pharmacokinetics and Pharmacodynamics

Ozanimod is an oral medication taken daily. It is readily absorbed in the gastrointestinal tract, reaching peak plasma concentrations between 8 and 24 h post administration [95]. The half-life is estimated to be around 21 h, with assumed active metabolites CC112273 and CC1084037 having a longer half-life of between 84 and 117 h [95,96]. The response is measured by the reduction in absolute lymphocyte count. Clearance of ozanimod is affected by body weight, with higher weights reducing effective clearance of metabolites, and smoking, which conversely increased the rate of clearance [97].

##### Therapeutic Drug Monitoring

Currently, there are no data on the role of therapeutic drug monitoring for ozanimod. There are methods to test for active metabolites allowing for therapeutic drug monitoring. The main concern with dose adjustments of ozanimod is that there are likely to be additive toxic effects of ozanimod at higher doses, mainly surrounding its negative chronotropic effects [98].

## 5. Discussion

Therapeutic drug monitoring may provide physicians with tools to achieve greater clinical efficacy with medications used in a more precise manner than empiric dosing of therapies. This is well established for treatments such as infliximab and adalimumab and provided means to proactively adjust therapies to achieve treatment goals.

In our review, the role of therapeutic drug monitoring in new biologic and small-molecule therapies is more contentious. For longer established biologic therapies, such as vedolizumab and ustekinumab, an exposure–response effect exists. However, there is no consensus on therapeutic drug targets, nor on the role of therapy dose adjustments in treatment based on trough drug levels. Previous studies have not only reported a wide variety of values, but the methodology in reporting has also varied, with some studies splitting groups into quartiles while others report absolute values. The wide variation in drug levels for achieving therapeutic response creates difficulty in discerning a clinically useful drug level to target. This variability may be further explained by differences in assays used in these studies, timing of drug level measurement and population characteristics, including biologic naivety and concurrent immunomodulator use. Current guidelines from the European Crohn’s and Colitis Organisation (ECCO) and the American Gastroenterological Association (AGA) do not provide an opinion on therapeutic drug monitoring for these agents [99,100,101]. So, whilst there is an exposure–response relationship for both vedolizumab and ustekinumab, the lack of consensus on approachable targets during the induction and maintenance phases makes it difficult to uniformly adapt clinical practice on the best method of using TDM with these agents.

To further improve the value of trough levels for ustekinumab and vedolizumab, further study is needed. Large randomised prospective trials where therapy is adjusted based on drug levels may provide further insight into the best method of using levels. This, however, may be difficult to achieve given that it is considered the best practice to optimise medical therapy in the setting of active disease, so trials will likely need to allow dose therapy adjustments in the active and control groups, which would make it more difficult to show a difference between groups. For example, the TAXIT study [2], which assessed the role of adjusting infliximab therapy based on trough concentrations versus clinical features, allowed all participants to reach therapeutic infliximab levels prior to randomisation. This may have resulted in higher remission rates in the control group and the lack of difference in remission rates noted between the groups. Disease relapses were lower in the concentration-guided group, so using TDM to adjust treatment may have been a more effective treatment strategy overall, despite the primary outcome being negative, which highlights the difficulty in designing a study to show this difference when both groups can adjust therapy.

For small-molecule therapies, JAK inhibitors and ozanimod, currently no commercially available drug monitoring tools exist. We predict that the role of therapeutic drug monitoring in this class of agents will be challenging. The pharmacokinetic properties of these agents, including short half-lives for JAK inhibitors, make it challenging to determine drug concentrations. Efficacy of these drugs may be measured by looking at end-target pathways, including flow cytometry methods into JAK, STAT phosphorylation for JAK inhibitors or active metabolite concentrations for ozanimod; however, no commercially available product is available. Furthermore, therapeutic drug monitoring may not provide any additional clinical benefit as escalation in dosing remains contentious due to the significant adverse events with higher doses, with small improvements in clinical response. More information on the role of adjusting therapy and measures of treatment levels will likely come with time and more use of the therapies in managing IBD.

## 6. Conclusions

While there appears to be an exposure–response correlation for ustekinumab and vedolizumab use in IBD, the role of drug levels to adjust therapy and target levels are less clear. The role of therapeutic drug monitoring in other advanced therapies that are not anti-TNF therapies remains unknown and further study is needed to adapt clinical practice to appropriately utilise therapeutic drug monitoring for these agents.

## Figures and Tables

**Table 1:** Methods for drug level testing.

	Methodology of Testing	Positives	Negatives
Enzyme-linked immunoassay (ELISA)	Uses antibodies to detect drug levels either via indirect or sandwich methodologies. Each method ultimately requires a specific antigen-bound antibody to detect the presence of the drug in serum samples. This is achieved via incubation, buffering and a coloured substrate solution being analysed via microplate reader.	Sensitive, specific and easy to use. Cost effective.	The presence of the drug in the serum can impact anti-drug antibody testing.
Radioimmunoassay	Specific drug-related radioactive antigen-bound antibodies are added to a medium along with patient serum. This is then centrifuged, and a gamma counter is used to determine drug levels and the presence of ADAs.	Rapid, highly sensitive and specific. No interference with the drug in the serum when detecting anti-drug antibodies.	Produces radioactive waste. Limited shelf-life due to radioactive decay.
Liquid-chromatography and mass spectrometry (LC-MS)	The patient serum sample is added to a dried blood spot card, dried, then removed and added to an internal standard solution and methanol base. The sample is then put through an extraction process through an ultrasonic bath and liquid extraction. The sample is dried and then undergoes mass spectrometry.	Sensitive and specific.	Time consuming.

## Abbreviations

ADA, anti-drug antibody; AGA, American Gastroenterological Association; anti-TNF, anti-tumour necrosis factor; CRP, C-reactive protein; ECCO, European Crohn’s and Colitis Organisation; ELISA, enzyme-linked immunoassay; IBD, inflammatory bowel disease; IL, interleukin; JAK, janus kinase; MAdCAM-1, mucosal vascular addressin cell adhesion molecule 1; MCP-1, monocyte chemoattractant protein-1; S1P, sphigosine-1-phosphate; STAT, signal transducer and activators of transcription; TDM, therapeutic drug monitoring.
